# Peripheral cutaneous synucleinopathy characteristics in genetic Parkinson’s disease

**DOI:** 10.3389/fneur.2024.1404492

**Published:** 2024-05-01

**Authors:** Yanpeng Yuan, Yangyang Wang, Minglei Liu, Haiyang Luo, Xiaojing Liu, Lanjun Li, Chengyuan Mao, Ting Yang, Shuo Li, Xiaoyun Zhang, Yuan Gao, Yuming Xu, Jing Yang

**Affiliations:** ^1^Department of Neurology, The First Affiliated Hospital of Zhengzhou University, Zhengzhou, Henan, China; ^2^Henan Key Laboratory of Cerebrovascular Diseases, Zhengzhou University, Zhengzhou, Henan, China; ^3^Institute of Neuroscience, Zhengzhou University, Zhengzhou, Henan, China; ^4^NHC Key Laboratory of Prevention and Treatment of Cerebrovascular Disease, Zhengzhou, Henan, China

**Keywords:** skin biopsy, genetic Parkinson’s disease, SAA, α-synuclein, *CHCHD2*, *RAB39B*

## Abstract

**Background:**

Cutaneous phosphorylated alpha-synuclein (p-α-syn) deposition is an important biomarker of idiopathic Parkinson’s disease (iPD). Recent studies have reported synucleinopathies in patients with common genetic forms of PD.

**Objective:**

This study aimed to detect p-α-syn deposition characteristic in rare genetic PD patients with *CHCHD2* or *RAB39B* mutations. Moreover, this study also aimed to describe peripheral alpha-synuclein prion-like activity in genetic PD patients, and acquire whether the cutaneous synucleinopathy characteristics of genetic PD are consistent with central neuropathologies.

**Methods:**

We performed four skin biopsy samples from the distal leg (DL) and proximal neck (C7) of 161 participants, including four patients with *CHCHD2* mutations, two patients with *RAB39B* mutations, 16 patients with *PRKN* mutations, 14 patients with *LRRK2* mutations, five patients with *GBA* mutations, 100 iPD patients, and 20 healthy controls. We detected cutaneous synucleinopathies using immunofluorescence staining and a seeding amplification assay (SAA). A systematic literature review was also conducted, involving 64 skin biopsies and 205 autopsies of genetic PD patients with synucleinopathy.

**Results:**

P-α-syn was deposited in the peripheral cutaneous nerves of PD patients with *CHCHD2*, *LRRK2*, or *GBA* mutations but not in those with *RAB39B* or *PRKN* mutations. There were no significant differences in the location or rate of α-syn-positive deposits between genetic PD and iPD patients. Peripheral cutaneous synucleinopathy appears to well represent brain synucleinopathy of genetic PD, especially autosomal dominant PD (AD-PD). Cutaneous α-synuclein SAA analysis of iPD and *LRRK2* and *GBA* mutation patients revealed prion-like activity.

**Conclusion:**

P-α-syn deposition in peripheral cutaneous nerves, detected using SAA and immunofluorescence staining, may serve as an accurate biomarker for genetic PD and iPD in the future.

## Introduction

Parkinson’s disease (PD) is a common neurodegenerative disorder caused by interactions among genetic and environmental risk factors (1). Approximately 15% of PD patients have a family history of the disease, with 5–10% presenting with a pattern of monogenic inheritance (2). The most common genetic risk factors for PD include *SNCA*, *LRRK2*, *PRKN*, *GBA*, and *DJ-1*, which are also associated with heterogeneous neuropathology and possibly with alpha-synuclein (α-syn)-positive Lewy body pathology (3–8). Cutaneous phosphorylated α-syn (p-α-syn) deposits have been detected in PD patients in recent years, highlighting their potential as biomarkers for premortem diagnosis (9). Cutaneous synucleinopathy has also been observed in PD patients with common genetic risk factors, including *SNCA*, *LRRK2*, *GBA*, and *DJ-1* (8, 10–13). Our research group previously identified two rare genes, *CHCHD2* T61I and *RAB39B* E179fsX48, in two Chinese families (14, 15). Features of synucleinopathy at autopsy have also been reported in patients harboring mutations at analogous sites (16–18). However, the presence of cutaneous synucleinopathy in PD patients with *CHCHD2* and *RAB39B* mutations has not yet to be determined.

Seeding amplification assay (SAA) were initially developed as specific and quantitative diagnostic tests for prion diseases (19). Recent applications have demonstrated the ability of SAA to detect the seeding activity of misfolded α-syn in the brain, cerebrospinal fluid (CSF), and skin samples from individuals with PD and Lewy body disease (LBD), achieving sensitivity rates of 92 to 95% and specificity rates of 98% to 100% (20–23). However, the efficacy of SAA in sensitively detecting α-syn prion-like activity in the skin of patients with genetic PD has not yet been established.

At present, substantia nigra pars compacta (SNpc) neuronal degeneration and Lewy body pathology within the brainstem remain critical hallmarks for the diagnosis of PD (1). Consequently, the identification of peripheral biomarkers of PD has emerged as a significant area of research interest. Several studies have shown that skin synucleinopathies in PD patients closely mirror intracranial pathologies, both in terms of morphology and main synuclein components (9, 24, 25), highlighting their considerable potential in peripheral biomarker research. To date, however, few studies have explored the characteristics of peripheral skin synucleinopathy in genetic PD, with no definitive evidence confirming the consistency between cutaneous and intracranial synucleinopathy alterations.

Hence, in the current study, we aimed to investigate cutaneous synucleinopathy in PD patients with *CHCHD2* and *RAB39B* mutations, explore α-syn prion-like activity in the skin of patients with genetic PD, and assess whether the peripheral cutaneous synucleinopathy observed in genetic PD is consistent with central neuropathology.

## Materials and methods

### Subjects and clinical assessment

A total of 161 participants were enrolled in this study. The cohort included four patients from one family with the *CHCHD2* T61I mutation; two patients from one family with the *RAB39B* E179fsX48 mutation; 16 patients with *PRKN* mutations; 14 patients with *LRRK2* mutations; five patients with *GBA* mutations; 100 idiopathic PD (iPD) patients with no known PD-associated mutations; and 20 healthy controls (Table 1). The two families with the *CHCHD2* T61I and *RAB39B* E179fsX48 mutations have been described in our previous studies (14, 15). All patients were recruited from the First Affiliated Hospital of Zhengzhou University (China) and fulfilled the Movement Disorder Society Clinical Diagnostic Criteria for PD (26). All participants underwent detailed neurological examination. Motor impairment was evaluated using the Movement Disorder Society Unified PD Rating Scale, part III (MDS-UPDRS-III) (27). Disease stage was assessed using the Hoehn & Yahr scale (28). Autonomic dysfunction and other nonmotor symptoms were evaluated using the nonmotor symptoms scale (NMSS) (29).

**Table 1:** Demographics and clinical characteristics of patients and healthy controls.

	*CHCHD2*	*RAB39B*	*PRKN*	*LRRK2*	*GBA*	iPD	HC
	(*n* = 4)	(*n* = 2)	(*n* = 16)	(*n* = 14)	(*n* = 5)	(*n* = 100)	(*n* = 20)
Age (y)	48.50 ± 12.79	43.50 ± 26.16	37.94 ± 8.59	61.79 ± 8.20	44.60 ± 10.58	64.07 ± 9.16	55.35 ± 9.03
Sex, male, *n* (%)	3 (75%)	2 (100%)	10 (62.50%)	4 (28.57%)	2(40%)	59 (59%)	10 (50%)
AAO (y)	41.75 ± 9.18	11.00 ± 1.41	28.06 ± 8.42	49.61 ± 7.14	41.20 ± 10.21	58.72 ± 10.07	/
Disease duration (y)	6.75 ± 4.11	32.00 ± 28.28	9.88 ± 6.53	7.72 ± 6.39	3.40 ± 2.30	5.35 ± 4.46	/
Hoehn and Yahr stage	1.63 ± 0.75	2.75 ± 0.35	2.41 ± 0.86	2.53 ± 0.50	2.00 ± 0.00	2.55 ± 0.97	/
MDS-UPDRS-III	21.25 ± 17.11	41.50 ± 23.33	32.88 ± 14.48	48.72 ± 28.16	23 ± 13.06	43.51 ± 19.18	/
NMSS score	71.25 ± 57.28	8.00 ± 0.00	13.69 ± 14.27	53.64 ± 22.74	16.00 ± 14.98	54.87 ± 46.51	/
RBD, *n* (%)	0 (0%)	1 (50%)	1 (6.67%)	8 (57.14%)	0(0%)	25 (25%)	0 (0%)
MMSE score	27.50 ± 3.00	19.00 ± 8.49	27.88 ± 1.59	26.47 ± 1.29	27.40 ± 0.89	26.70 ± 3.25	27.35 ± 2.08

*CHCHD2*, coiled-coil-helix-coil-helix domain containing 2; *RAB39B*, Ras analog in brain 39b; *GBA*, glucocerebrosidase; *PRKN*, Parkin RBR E3 ubiquitin protein ligase; *LRRK2*, leucine-rich repeat kinase 2; iPD, idiopathic Parkinson’s disease; HC, healthy control; AAO, age at onset; MDS-UPDRS III, Movement Disorder Society Unified Parkinson’s Disease Ranking Scale part III; NMSS, nonmotor symptoms scale; RBD, rapid eye movement sleep behavior disorder; MMSE, Mini-Mental State Examination Scale.

This study was approved by the Ethics Committee of the First Affiliated Hospital of Zhengzhou University (2019-KY-294). All work was carried out in accordance with the Code of Ethics of the World Medical Association (Declaration of Helsinki) for experiments involving humans. All participants provided written informed consent to participate.

### Skin biopsy

Skin punch biopsies (3 mm in diameter, four samples) were taken from the distal leg (DL) (two samples) and proximal neck region (C7) (two samples) of the 161 participants under 20 mg/mL lidocaine local anesthesia using a sterile technique according to current guidelines (9). The two samples were parallel. There were 5 mm distance between two samples. The four obtained biopsies included the epidermis and subpapillary dermis. Biopsy specimens were immediately immersion-fixed with Zamboni solution (G2190; Solarbio, China) for 12–24 h. The fixation buffer was changed to tissue cryoprotectant solution until use. The biopsy tissues were sliced into 50-μm sections using a frozen slicer (Leica CM1950, Mannheim, Germany).

### Brain autopsy cases

A systematic literature review of brain autopsy cases was conducted using the terms “autopsy,” “genetic Parkinson’s disease,” “α-synuclein,” “Lewy body pathology,” “brain pathology,” and “synucleinopathy,” as well as specific gene nomenclature (*CHCHD2*, *RAB39B*, *PRKN/PARK2/Parkin*, *LRRK2*, *GBA*, *SNCA*, and *DJ-1*), and any combination of the above.

### Immunofluorescence staining

Free-floating immunofluorescence was performed on one of every five 50-μm thick serial sections of skin biopsies, as described previously (13). To evaluate the intra-axonal localization of p-α-syn deposits, four additional 50 μm thickness sections at 200 μm interval from each skin samples, double-labeling immunofluorescence analysis of all patients and controls was conducted using the anti-protein-encoding gene product 9.5 (PGP9.5), anti-p-α-syn (p-syn), anti-calcitonin gene-related peptide (CGRP), anti-tyrosine hydroxylase (TH), anti-vasoactive intestinal peptide (VIP), anti-α-syn, anti-5G4, anti-ASyO5, anti-AT8, anti-HT7, anti-TDP-43, anti-ubiquitin, anti-Aβ40, and anti-Aβ42 (see 1Supplementary Table S1 for detailed antibody information). The secondary antibodies used included Alexa Fluor 594-conjugated goat anti-mouse/rabbit immunoglobulin G (IgG) (Origene, China, 1:400) and Alexa Fluor 488-conjugated goat anti-rabbit/mouse IgG (Origene, China, 1:800). Photomicrographs were taken using a confocal laser scanning microscope (Nikon, A1 HD25, Japan) and fluorescence microscope (Nikon, Eclipse Ni, Japan).

The immunostaining should be repeated when negative signal was found for the first time. The immunostaining results were considered “positive” when PGP9.5 was colocalized with anti-p-α-syn, anti-ASyO5, anti-5G4, anti-α-synuclein, anti-AT8, anti-HT7, anti-TDP-43, anti-ubiquitin, anti-Aβ40, or anti-Aβ42 in skin biopsy nerve fibers. The immunostaining results were also considered “positive” when p-syn was colocalized with anti-CGRP, anti-TH and anti-VIP in skin biopsy nerve fibers.

### Preparation of skin tissues for SAA

Skin tissue preparation was conducted as described previously (22). Briefly, skin tissues were washed three times in 1× Tris (hydroxymethyl) aminomethane-buffered saline and chopped into small pieces. Skin homogenates (10%, weight/volume) were prepared in skin lysis buffer containing 2 mmol of calcium chloride and 0.25% (weight/volume) collagenase A (Roche, 10,103,586,001, Germany) in Tris-buffered saline and incubated in a shaker at 37°C for 4 h, followed by homogenization in a Mini-Beadbeater (BioSpec; Laboratory Supply Network) for 1 min. After sonication to disrupt the remaining tissue structures, the samples were centrifuged for 5 min at 500 × *g* for collection of the supernatant fraction.

### Seeding amplification assay

The SAA was conducted as described previously (22), with some modifications. Briefly, the SAA reaction mixture was composed of 40 mM phosphate buffer (pH 8.0), 170 mM NaCl, 0.1 mg/mL recombinant human wild-type α-syn, 10 μM thioflavinT (ThT) (Sigma, T3516-5G, Germany), and 0.00125% sodium dodecyl sulfate. Reaction mixture aliquots (98 μL) were loaded into each well of a black 96-well plate with a clear bottom (Nunc) preloaded with 800 μm Silica beads (OPS Diagnostics, 800-200-01, United States), with the cells then seeded with 2 μL of skin homogenate.

### Statistical analysis

All data are expressed as means ± standard deviation (SD) or percentages. All statistical analyses were carried out using GraphPad Prism 6 (GraphPad Software, La Jolla California, United States). Independent sample *t*-test or Mann–Whitney U test was used to analyze continuous variables and chi-square test was used to analyze categorical variables. Differences were considered statistically significant at *p* < 0.05.

## Results

### Genetic and clinical features of genetic PD patients

The *CHCHD2* T61I mutation was identified in a family with autosomal dominant PD. Individuals with this mutation exhibited typical parkinsonism, although one patient only exhibited simple tremor. The mean age at onset (AAO) was 39.33 ± 8.50 years, and the mean disease duration was 4.67 ± 3.21 years (Tables 1, 2). Patients exhibited mild motor symptoms but severe nonmotor symptoms, including orthostatic hypotension, constipation, depression, smell impairment, and sexual dysfunction. A positive response to levodopa treatment was observed in all four patients.

**Table 2:** Genetic and clinical features of patients with genetic PD.

Gene	Mutation sites	Sex	Age (y)	AAO (y)	Initial symptoms	Disease duration (y)	H&Y stage	MDS-UPDRS III score	NMSS score	RBD
*CHCHD2*	p. T61I	M	54	48	Resting tremor	6	2	28	135	−
*CHCHD2*	p. T61I	M	46	39	Essential tremor	7	1	4	28	−
*CHCHD2*	p. T61I	M	32	30	Resting tremor	2	1	5	30	−
*CHCHD2*	p. T61I	F	62	50	Resting tremor	12	2.5	42	104	−
*RAB39B*	p. E179fsX48	M	24	12	Resting tremor	12	2.5	25	8	−
*RAB39B*	p. E179fsX48	M	62	10	Resting tremor	52	3	58	8	+
*PRKN*	E3-4 del; p. G284R	F	31	21	Bradykinesia-rigidity	10	3	47	59	+
*PRKN*	E4 del; p. G284R	M	40	27	Bradykinesia-rigidity	13	2	18	8	−
*PRKN*	E6-7 del; p.E309*	M	26	11	Bradykinesia-rigidity	15	2	39	3	−
*PRKN*	E2-3 del; c.2T>C	F	42	36	Resting tremor	6	3	36	36	−
*PRKN*	E3 del; E6 del	F	41	36	Bradykinesia-rigidity	5	2	14	7	−
*PRKN*	E3 del; E7 del	M	47	37	Bradykinesia	10	3	65	17	−
*PRKN*	E3 del; E5 del	M	29	25	Resting tremor	4	2.5	41	8	−
*PRKN*	E3 del; c.1310delC	F	29	26	Rigidity	3	3	26	10	−
*PRKN*	E2-4 dup	F	36	20	Bradykinesia-rigidity	16	2	38	21	−
*PRKN*	p.S223*; E2 del	M	28	20	Resting tremor-Rigidity	8	2	26	6	−
*PRKN*	c. 933 + 5G›T Homo	F	57	31	Bradykinesia	26	3	37	18	−
*PRKN*	E3 del; E4 del	M	44	36	Resting tremor	8	2	15	2	−
*PRKN*	p.S286 Profs*12; E3 duplication	M	48	33	Resting tremor	15	2.5	54	0	−
*PRKN*	splice-3, E3-4 del	M	32	31	Bradykinesia	1	2	15	6	−
*PRKN*	E3-4 del, E7 del	M	33	17	Bradykinesia	16	2	22	29	−
*PRKN*	E5-6 dup, c.2T>C	M	44	41	Bradykinesia	3	2	35	8	−
*GBA*	p.L483P	F	32	25	Resting tremor	7	2	34	1	−
*GBA*	p.A502C	M	42	38	Bradykinesia	4	2	13	3	−
*GBA*	p.A502H	M	52	51	Bradykinesia	1	2	22	20	−
*GBA*	p.L483P	F	49	47	Resting tremor	2	2	9	18	−
*GBA*	p.A123S	M	43	38	Resting tremor	5	2	39	38	−
*LRRK2*	p. G2385R	M	64	59	Resting tremor	5	2.5	28	19	−
*LRRK2*	p. G2385R	M	65	63	Bradykinesia	2	2.5	42	73	+
*LRRK2*	p. G2385R	M	46	45	Resting tremor	1	1	15	37	−
*LRRK2*	p. G2385R	M	54	48	Bradykinesia	6	3	64	73	+
*LRRK2*	p. G2385R	M	74	73	Bradykinesia	1	2	23	34	−
*LRRK2*	p. G2385R	F	65	61	Bradykinesia	4	2	41	60	+
*LRRK2*	p. G2385R	M	75	71	Resting tremor	4	2.5	30	34	+
*LRRK2*	p. G2385R	M	61	56	Resting tremor	5	4	69	72	+
*LRRK2*	p. G2385R	F	58	43	Resting tremor	15	3	80	59	+
*LRRK2*	p. G2385R	M	64	61	Resting tremor-Bradykinesia	3	2.5	54	56	+
*LRRK2*	p. G2385R	F	63	59	Resting tremor-Bradykinesia	4	3	89	86	−
*LRRK2*	p. G2385R	F	67	55	Resting tremor	12	3	91	80	+
*LRRK2*	p. G2385R	F	60	45	Resting tremor	15	3	75	25	−
*LRRK2*	p. A1728H	F	50	47	Bradykinesia	3	2	19	14	−

*CHCHD2*, coiled-coil-helix-coil-helix domain containing 2; *RAB39B*, Ras analog in brain 39b; *PRKN*, parkin RBR E3 ubiquitin protein ligase; *LRRK2*, leucine-rich repeat kinase 2; *GBA*, glucocerebrosidase; AAO, age at onset; MDS-UPDRS III, Movement Disorder Society Unified Parkinson’s Disease Ranking Scale part III; NMSS, nonmotor symptoms scale; RBD, rapid eye movement sleep behavior disorder.

The *RAB39B* E179fsX48 mutation was identified in a family with X-linked PD, with both patients presenting with juvenile parkinsonism. The mean AAO was 11.00 ± 1.41 years (Table 1). Both patients exhibited major motor symptoms but mild nonmotor symptoms, while one patient exhibited moderate cognitive decline. Disease progression was extremely slow, with a mean disease duration of 32.00 ± 28.28 years (Tables 1, 2) and both patients showed moderate responses to levodopa treatment. In addition, compound heterozygous *PRKN* mutations were identified in 15 unrelated patients, with a single case of homozygosity found in a patient resulting from a consanguineous marriage. These patients presented with early-onset parkinsonism and slow progression, exhibiting a mean AAO of 28.06 ± 8.42 years and mean disease duration of 9.88 ± 6.53 years (Tables 1, 2). All 16 patients responded well to levodopa treatment, although three patients experienced dyskinesias as a side effect.

Thirteen unrelated patients carried the genetic risk factor *LRRK2* G2385R, while one harbored the A1728H mutation. These patients presented with iPD, with a mean AAO of 49.61 ± 7.14 years and mean disease duration of 7.72 ± 6.39 years (Tables 1, 2). Twelve out of the 14 patients responded well to levodopa therapy.

Four out of five patients with heterozygous *GBA* mutations exhibited early-onset parkinsonism, with a mean AAO of 41.20 ± 10.21 years and mean disease duration of 3.40 ± 2.30 years (Tables 1, 2). Five patients responded well to levodopa treatment, although two developed dyskinesias.

### Similar p-α-syn deposits in cutaneous nerve fibers in genetic PD and iPD patients

**Figure 1:**
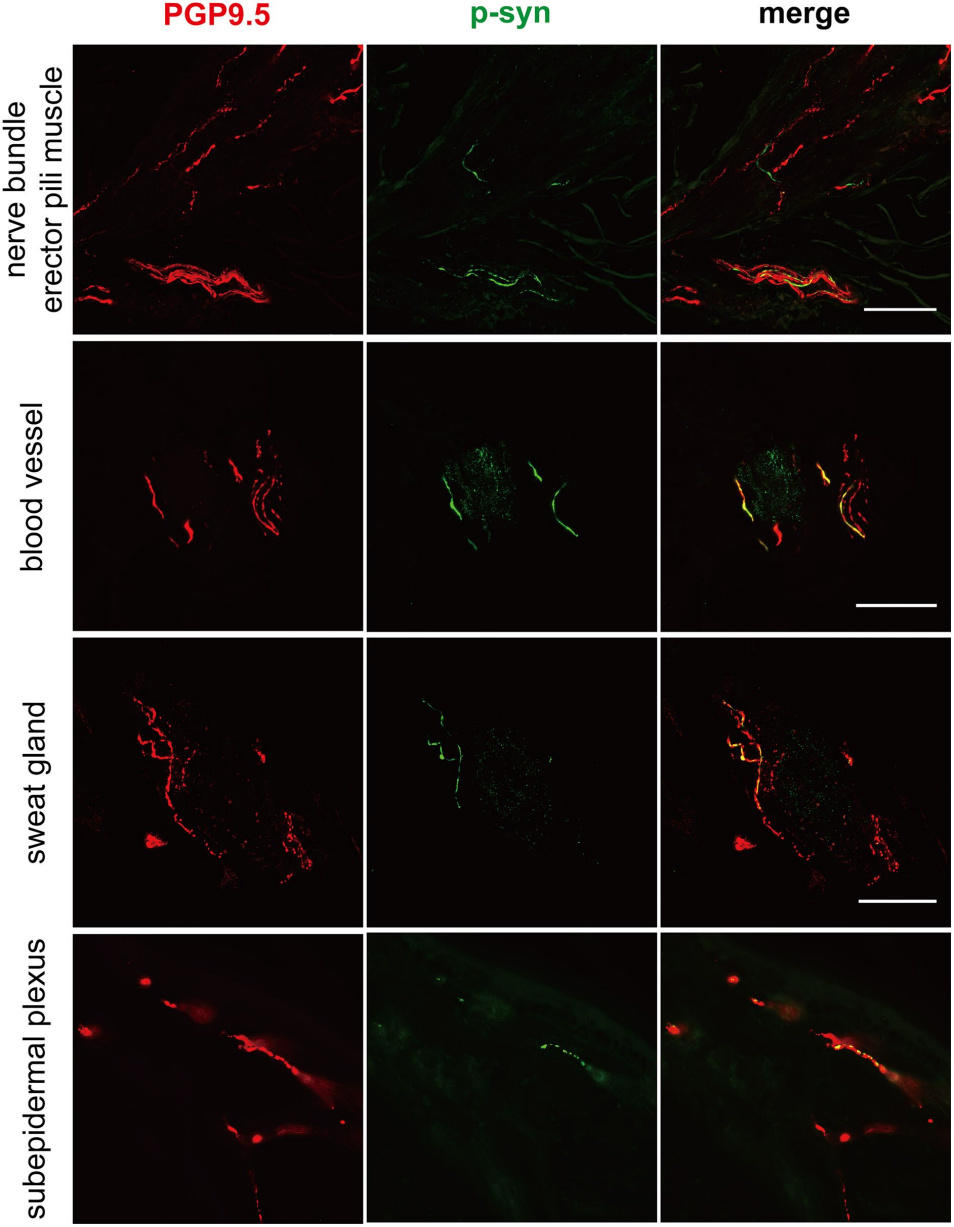
Immunofluorescence of different skin biopsy structures from PD patients with *CHCHD2*, *LRRK2* and *GBA* mutations. Phosphorylated α-synuclein (p-α-syn, green) was deposited in dermal nerve bundles, erector pili muscles, blood vessels, sweat glands, and subepidermal plexuses. PGP9.5 (red): protein-coding gene product 9.5. Scale bar = 50 μm.

P-α-syn deposits were detected in the subepidermal plexus, dermal nerve bundles, arrector pili muscles, and blood vessels (Figure 1) of three PD patients with the *CHCHD2* T61I mutation (75%), 11 PD patients with *LRRK2* mutations (78.57%), and two patients with *GBA* mutations (40%; Table 3). However, p-α-syn were not detected in PD patients with *RAB39B* (0%, 0/2) and *PRKN* (0%, 0/16) mutations.

**Table 3:** Summary of differences in p-α-syn-positivity ratio in skin biopsies between genetic PD and iPD patients.

	*CHCHD2*	*LRRK2*	*GBA*	iPD	*p-*value		
	(*n* = 4)	(*n* = 14)	(*n* = 5)	(*n* = 100)	a	b	c
p-α-syn-positive ratio, *n* (%)	3 (75%)	11 (78.57%)	2 (40%)	72 (72%)	1.00^3^	0.62^2^	0.15^2^
Subepidermal plexus, *n* (%)	2 (50%)	7 (50%)	0 (0%)	52 (52%)	1.00^3^	0.68^1^	0.06^3^
Dermal bundle, *n* (%)	3 (75%)	9 (64.29%)	2 (40%)	65 (65%)	1.00^2^	1.00^2^	0.35^2^
Sweat gland, *n* (%)	0 (0%)	0 (0%)	0 (0%)	9 (9%)	1.00^3^	1.00^3^	1.00^3^
Arrector muscle pili, *n* (%)	1 (25%)	1 (7.14%)	2 (40%)	6 (6%)	0.19^2^	0.56^2^	0.26^2^
Vessel, *n* (%)	0 (0%)	3 (21.43%)	0 (0%)	30 (30%)	0.55^3^	0.53^2^	0.55^3^
Follicle *n* (%)	0 (0%)	0 (0%)	0 (0%)	2 (2%)	1.00^3^	1.00^3^	1.00^3^
p-α-syn-positive in DL, *n* (%)	3 (75%)	8 (57.14%)	2 (40%)	52 (52%)	1.00^2^	0.68^1^	0.67^2^
p-α-syn-positive in C7, *n* (%)	3 (75%)	9 (64.29%)	0 (0%)	57 (57%)	1.00^2^	0.81^1^	0.01^3^ *

*P*-values are shown as follows: ^a^*CHCHD2* vs. iPD; ^b^*LRRK2* vs. iPD; ^c^*GBA* vs. iPD. ^*^p < 0.05. DL, distal leg. ^1^Pearson χ^2^ (*n* ≥ 40 and T ≥ 5). ^2^Continuous correction (*n* ≥ 40 and 1 ≤ T ≤ 5). ^3^Fisher’s exact probabilities (*n* < 40 or T < 1).

**Figure 2:**
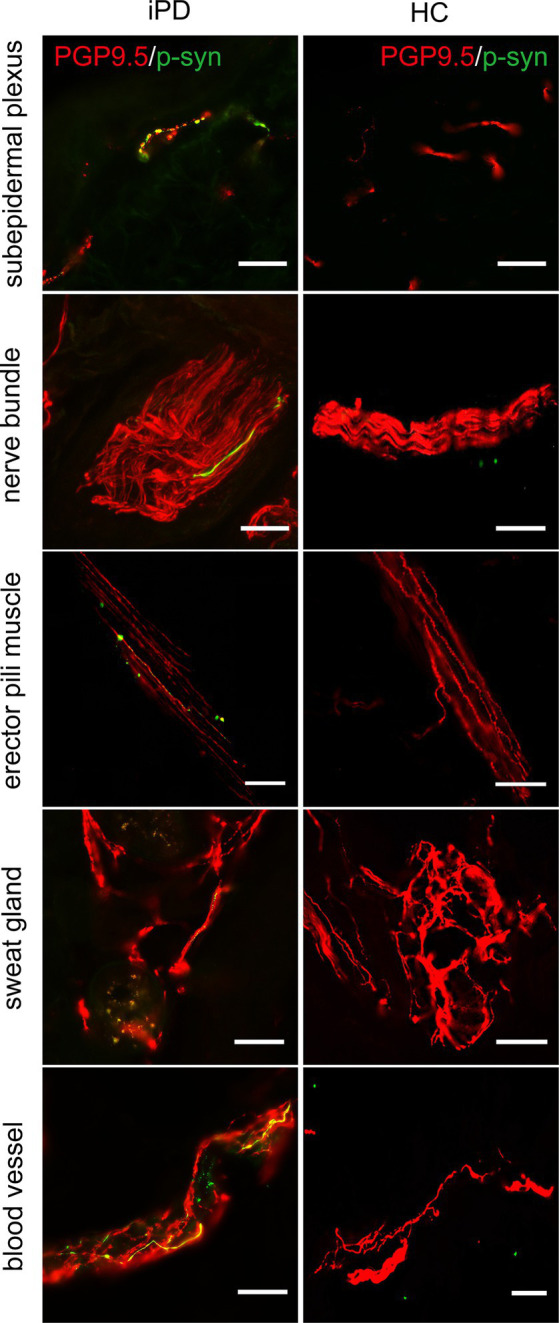
Immunofluorescence staining of different skin biopsy structures from iPD patients and healthy controls. Phosphorylated α-synuclein (p-α-syn, green) was deposited in subepidermal plexus, dermal nerve bundle, erector pili muscle, sweat gland, and blood vessels. PGP9.5 (red): protein-coding gene product 9.5. Scale bar = 50 μm.

Further analysis of p-α-syn deposition in the 100 iPD patients and 20 healthy controls revealed that p-α-syn was deposited in 72/100 (72%) iPD patients (Table 3) but not in any of the healthy controls. Furthermore, p-α-syn-positive nerve fibers were found in the subepidermal plexus, dermal nerve bundles, arrector pili muscles, sweat glands, and blood vessels (Figure 2).

**Figure 3:**
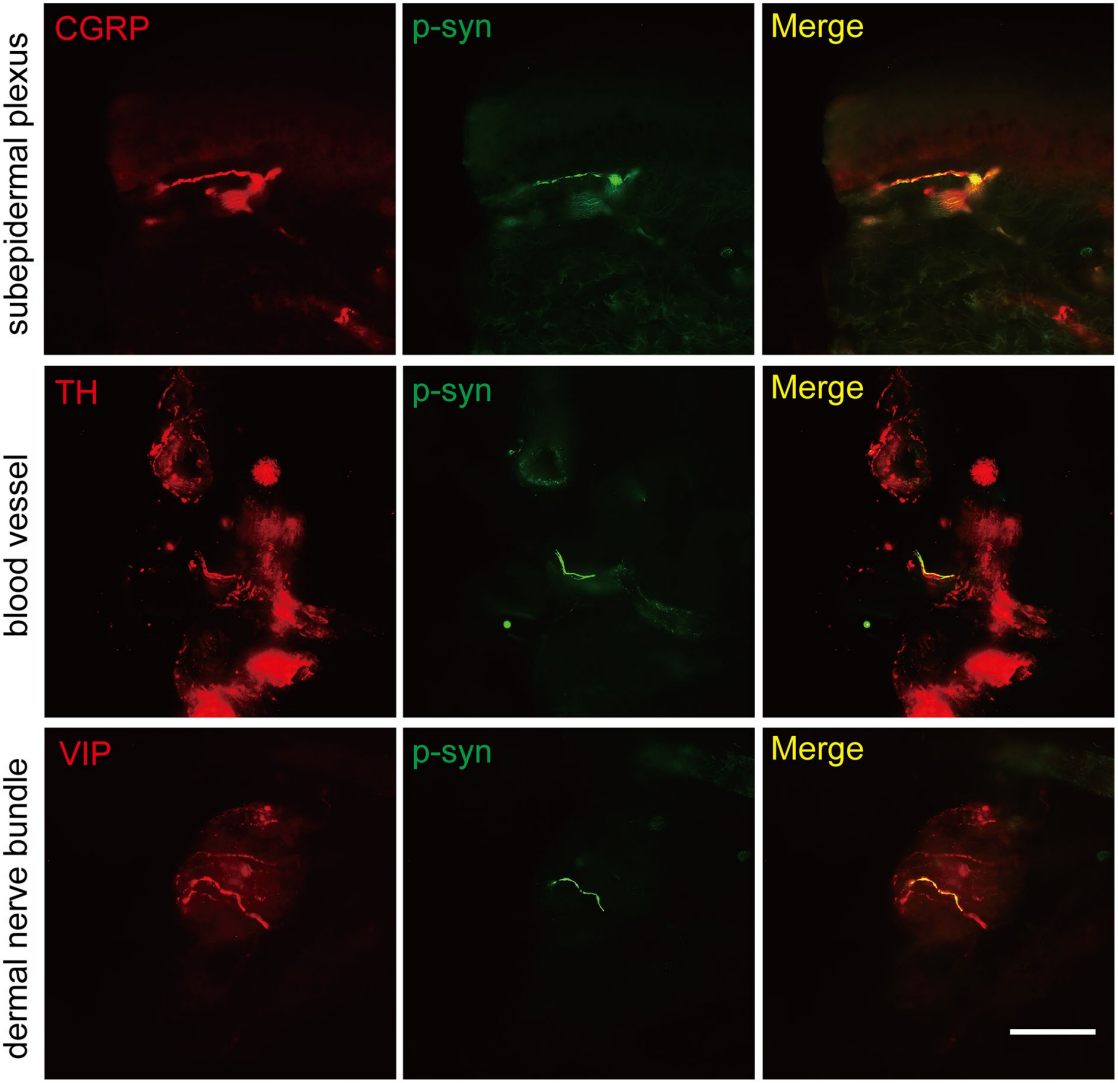
Immunofluorescence staining of somatosensory and autonomic nerve structures of PD patients. Somatosensory nerve antibody calcitonin gene-related peptide (CGRP, red) was double-stained in subepidermal plexus, sympathetic nerve antibody tyrosine hydroxylase (TH, red) was positive in blood vessels, and parasympathetic nerve antibody vasoactive intestinal peptide (VIP, red) was double-stained in dermal nerve bundles. Phosphorylated α-synuclein (p-α-syn, green). Scale bar = 50 μm.

The somatosensory nerve antibody CGRP, sympathetic nerve antibody TH, and parasympathetic nerve antibody VIP were used to identify p-α-syn-positive structures. Results showed that CGRP was positive in the subepidermal plexus (Figure 3) and some superficial dermal nerve bundles. TH was positive in the arrector pili muscles, sweat glands, blood vessels (Figure 3), and some deep dermal nerve bundles. VIP was positive in the sudomotor fibers and some deep dermal nerve bundles (Figure 3).

### Similar p-α-syn deposition rates and sites in skin biopsies from genetic PD and iPD patients

The total p-α-syn-positive ratios were 3/4 (75%) in the *CHCHD2* group, 11/14 (78.57%) in the *LRRK2* group, 2/5 (40%) in the *GBA* group, and 72/100 (72%) in the iPD group. No significant differences in skin p-α-syn-positive deposition were observed among the *CHCHD2*, *LRRK2*, *GBA*, and iPD patients (*p* = 1.00, *p* = 0.62, *p* = 0.15; Table 3). The skin biopsy innervation structures included the subepidermal plexus, dermal nerve bundles, arrector pili muscles, sweat glands, blood vessels, and follicles. The p-α-syn-positive ratios for the subepidermal plexus were 2/4 (50%) in the *CHCHD2* group, 7/14 (50%) in the *LRRK2* group, and 52/100 (52%) in the iPD group (*p* = 1.00, *p* = 1.00). The p-α-syn-positive ratios for the dermal nerve bundles were 3/4 (75%) in the *CHCHD2* group, 9/14 (64.29%) in the *LRRK2* group, 2/5 (40%) in the *GBA* group, and 65/100 (65%) in the iPD group (*p* = 1.00, *p* = 1.00 and *p* = 0.35). The p-α-syn-positive ratios for the arrector pili muscles were 1/4 (25%) in the *CHCHD2* group, 1/12 (7.14%) in the *LRRK2* group, 2/5 (40%) in the *GBA* group, and 6/100 (6%) in the iPD group (*p* = 0.19, *p* = 0.56 and *p* = 0.26). No significant differences in p-α-syn-positive deposition were found in the different skin biopsy innervation structures between the genetic PD and iPD patients (Table 3).

The p-α-syn-positive percentages were 3/4 (75%) in the DL and C7 of the *CHCHD2* group, 8/14 (57.14%) in the DL and 9/14 (64.29%) in the C7 of the *LRRK2* group, 2/5 (40%) in the DL of the *GBA* group, and 52/100 (52%) in the DL and 57/100 (57%) in the C7 of the iPD group. While no significant differences were observed in the percentage of p-α-syn-positive cells in the DL (*p* = 1.00, *p* = 0.68, and *p* = 0.67), significant differences were found between the *GBA* and iPD groups in the C7 (*p* = 0.01; Table 3).

### Skin biopsy and brain autopsy synucleinopathy results from our data and literature review data were highly consistent with those of genetic PD patients

We examined 41 skin biopsies from genetic PD patients (*CHCHD2*, *n* = 4; *RAB39B*, *n* = 2; *GBA*, *n* = 5; *PRKN*, *n* = 16; and *LRRK2*, *n* = 14) and retrieved 64 skin biopsy and 205 brain autopsy genetic PD patients from the literature review (Table 4). The skin biopsy synucleinopathies of the *CHCHD2*, *RAB39B*, *LRRK2*, and *GBA* groups were the same as the brain autopsy synucleinopathies. However, there was a significant difference in synucleinopathies between the PRKN-related PD patients and brain autopsies (Table 4).

**Table 4:** Summary of α-syn deposition rate in skin biopsies and brain autopsies of genetic PD patients.

Gene	Skin biopsy		Autopsy	*P*-value
	(Our data *n* = 41)	(Data from literature *n* = 64)	(Data from literature *n* = 205)	
*CHCHD2*	75% (3/4)	/	100% (1/1)	1.00^3^
*RAB39B*	0% (0/2)	/	100% (1/1)	0.33^3^
*LRRK2*	78.57% (11/14)	100% (7/7)	64.4% (38/59)	0.10^2^
*PRKN*	0% (0/16)	0% (0/20)	55% (11/20)	<0.0001^3*^
*GBA*	40% (2/5)	64% (16/25)	96% (92/96)	0.10^1^
*SNCA*	/	100% (10/10)	100% (27/27)	1.00^3^
*DJ-1*	/	100% (2/2)	100% (1/1)	1.00^3^

^1^Pearson χ^2^ (*n* ≥ 40 and T ≥ 5); ^2^Continuous correction (*n* ≥ 40 and 1 ≤ T ≤ 5); ^3^Fisher’s exact probabilities (*n* < 40 or T < 1). ^*^p < 0.05.

The detailed skin biopsy and brain autopsy synucleinopathy results for the genetic PD patients are shown in Table 5. Mendelian inheritance includes autosomal dominant (AD), autosomal recessive (AR), X-linked dominant (XD), and X-linked recessive inheritance (XR). Here, *CHCHD2*, *LRRK2*, and *GBA* were recognized as AD-PD-related genes. Skin biopsies from one family with the *CHCHD2* T61I mutation were positive for p-α-syn deposits. One brain autopsy patient with the *CHCHD2* T61I mutation showed p-α-syn-positive deposits, including diffuse Lewy bodies (LBs) and Lewy neurites (LNs). Eleven out of 14 *LRRK2* mutation carriers contained p-α-syn-positive deposits in their skin biopsies. Based on our literature review, *LRRK2* G2019S/R1441G/R1441C mutations were also found in European and North American individuals with p-α-syn-positive deposition in skin biopsies and brain autopsies. Two out of the five *GBA* mutation carriers contained p-α-syn-positive deposits in their skin biopsies. The autopsy *GBA* mutation sites were primarily distributed in Europe and North America. Notably, p-α-syn depositions were positive in skin biopsies and brain autopsy samples in patients harboring *GBA* mutations.

**Table 5:** Summary of observed α-syn deposition in skin biopsies and brain autopsies of patients with genetic PD.

Gene	Mode of inheritance	Skin biopsy			Autopsy		
		Mutation sites	Ethnicity	α-syn deposition	Mutation sites	Ethnicity	α-syn deposition
*CHCHD2*	AD	T61I (our data)	Asian	p-α-syn (+)	T61I (16)	Asian	p-α-syn (+)
*LRRK2*	AD	G2385R; A1728H (our data); G2019S (30)	Asian; North American	p-α-syn (+)	G2019S; (1, 3, 31–35) R1441G; (36) R1441C; (37); others; (38, 39)	European; North American	p-α-syn (+)
		G2019S, R1441G (30)					
*SNCA*	AD	E46K; (8) A53T; Duplication (30)	European; North American	p-α-syn (+)	E46K; (40) A53T; (41–44) Duplication; (45–49) A30P; (50) others (49, 51)	European; North American	Alpha-syn (+)
*GBA*	AD	L483P; A502C; A502H; A123S (our data); N370S; E326K; L444P; (10) N409S; L483P; N409S homo (30)	Asian; European; North American	p-α-syn (+)	N370S; E326K; L444P; D409H; R496H; N370S homo; et al. (4, 5, 52–61)	European; North American	Alpha-syn (+)
*DJ-1*	AR	E4 del; A36Cfs*12 (12) E3 del, A35Cfs*12 (30)	North American	Alpha-syn (+)	L172Q homo (62)	European	Alpha-syn (+)
*PRKN*	AR	E4 del, G284R; E3-4 del, G284R; E6-7 del, E309*; E2-3 del, c.2T>C; E3 del, E6 del; E3 del, E7 del; E3 del, E5 del; E3 del, c. 1310delC; E2-4 dup; c.933+5G>T homo; p.S223*, E2 del; E3 del, E4 del; S286 Profs*12, E3 dup; E3-4 del, E7 del; splice-3, E3-4 del; E5-6 dup, c.2T>C (Above all our data) R275 W, E3-4 del; c.7+5G>T, E3-4 del; p. Asn52fs homo; E3 del, E3-4 del; (30) E7 del, V56E (12, 30); E2 del, E5 del (11); E11 del, G429D; T415N homo (12, 30)	Asian; North American; European	p-α-syn (−)	R275W (63–65); R275 W, E6 del; R275 W, G430 W; R275 W, Pro113fs; G430D, Pro113fs; (66); E7 del, 1,072 T del (6); E3 del homo (67); E2-4 del homo (7); E2-4 dup, E3-4 del; E4 del homo; E6-7 dup homo; E10-11 dup homo; C431F homo; C431F, E2-4 dup; E2 trip, E2-3 del; (68)	European; North American; Asian	p-α-syn (+)
*RAB39B*	XD	E179fsX48 (our data)	Asian	p-α-syn (−)	T168K (17)	Australian	Alpha-syn (+)

Red Mutation sites from skin biopsies were obtained in our study, while the remaining skin biopsy and autopsy data were acquired from literature review.

The *PRKN* gene is associated with AR-PD. In this study, we identified 15 PD patients with compound heterozygous *PRKN* mutation and one patient with homozygous *PRKN* mutation. Based on a review of the literature on skin biopsies literature, we retrieved 18 compound heterozygous *PRKN* mutations in North American and European PD patients. Skin biopsies from all 34 patients with *PRKN* mutations were negative for p-α-syn deposits. Furthermore, a review of autopsy literature identified 20 patients with either homozygous or compound heterozygous *PRKN* mutations, mostly from Europe and North America. Although the *PRKN* mutation sites differed between skin biopsy and brain autopsy samples, 55% (11/20, no Asian patients) of brain autopsies showed α-syn-positive deposition.

One PD family with the *RAB39B* E179fsX48 mutation, which exhibited XD inheritance, was sampled. The skin biopsies of both patients were negative for p-α-syn deposition. However, brain autopsy samples of an Australian PD patient carrying the *RAB39B* T168K mutation revealed p-α-syn-positive deposits in the SN and cortex.

### Co-neuropathology in skin biopsy of genetic PD and iPD patients

**Figure 4:**
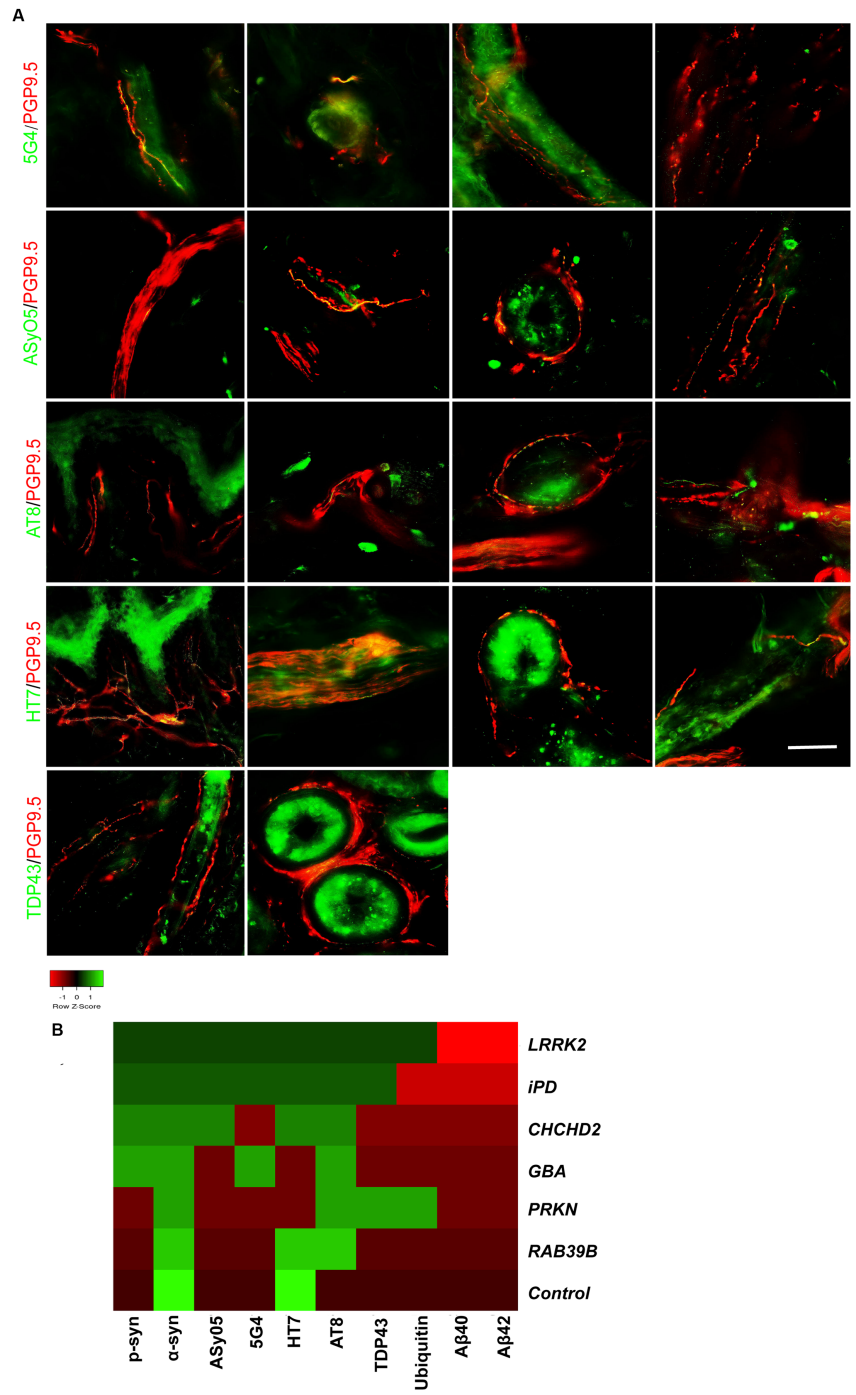
Co-neuropathology in patients with genetic PD and iPD. **(A)** Immunofluorescence staining results. Aggregated α-syn (5G4, green) was deposited in dermal nerve bundles, blood vessels, sweat glands, and erector pili muscles. Oligomer-specific α-syn (ASyO5, green) was deposited in dermal nerve bundles, blood vessels, sweat glands, and erector pili muscles. Phosphorylated tau (AT8, green) was deposited in subepidermal plexus, dermal nerve bundle, sweat glands, and blood vessels. Tau (HT7, green) was deposited in subepidermal plexus, dermal nerve bundles, sweat glands, and blood vessels. TAR DNA-binding protein 43 (TDP-43, green) was deposited in erector pili muscles and sweat glands. PGP9.5 (red): protein-coding gene product 9.5. Scale bar = 50 μm. **(B)** Heatmap results of co-neuropathology. Red indicates positive deposition, green indicates negative deposition. P-α-syn, α-syn, ASyO5, 5G4 indicate synucleinopathy, HT7, AT8 indicate tauopathy, and Aβ40 and Aβ42 indicate amyloid pathology. Other pathology contains TDP-43 and ubiquitin. Genetic PD patients and iPD patients exhibited heterogeneous co-neuropathology.

Co-neuropathology analysis of skin biopsies from patients with genetic PD and iPD was also performed. Immunofluorescence staining revealed aggregated α-syn (5G4 and ASyO5, green) deposits in dermal nerve bundles, blood vessels, sweat glands, and erector pili muscles (Figure 4A, from left to right). Tauopathies (AT8 and HT7, green) were deposited in the subepidermal plexus, dermal nerve bundles, sweat glands, and blood vessels (Figure 4A). TAR DNA-binding protein 43 (TDP-43, green) was deposited in erector pili muscles and sweat glands (Figure 4A).

Patients carrying *LRRK2*, *CHCHD2*, and *GBA* mutations, as well as those with iPD, displayed similar co-neuropathologies, including positivity for p-α-syn, α-syn, ASyO5, 5G4 (synucleinopathy), and HT7 and AT8 (p-tauopathy). PD patients with *PRKN* and *RAB39B* mutations commonly exhibited α-syn (synucleinopathy) and AT8 (p-tau) positivity. PD patients with *LRRK2* and *PRKN* mutations also exhibited TDP-43 and ubiquitin positivity (Figure 4B).

### Cutaneous α-synuclein exhibited prion-like activity in genetic PD and iPD

**Figure 5:**
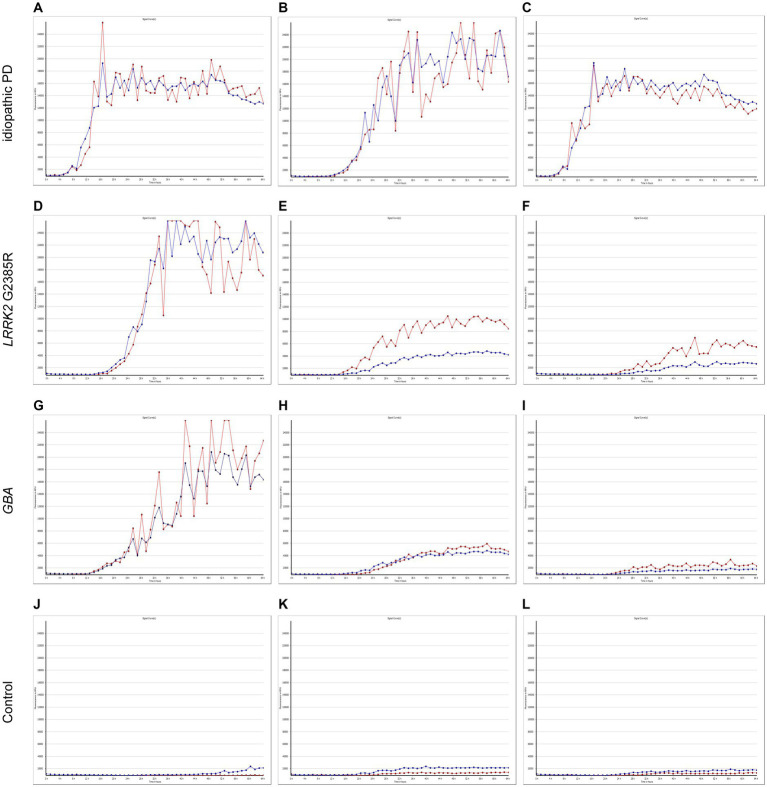
SAA curves of patients with genetic PD and iPD. α-Synuclein SAA curves of three idiopathic PD patients **(A–C)**, three *LRRK2* G2385R mutation patients **(D–F)**, three *GBA* mutation patients **(G–I)** and three healthy controls **(J–L)**. The red and blue lines shown in each figure represent replicates from the same subject.

The α-syn RT-QuIC curves of iPD patients (Figures 5A–C), PD patients with *LRRK2* G2385R mutations (Figures 5D–F), PD patients with *GBA* mutations (Figures 5G–I), and healthy control subjects (Figures 5J–L) are shown. Three iPD patients (Figures 5A–C), including one with the *LRRK2* G2385R mutation (Figure 5D) and one with the *GBA* A502H mutation (Figure 5G), exhibited RT-QuIC-positive curves. As shown in Table 6, α-syn RT-QuIC can identify iPD-, *LRRK2-*, and *GBA*-positive synucleinopathies with 100% sensitivity and 100% specificity in skin biopsies.

**Table 6:** α-Syn SAA diagnostic values in skin biopsies from patients with genetic PD.

	*N*	SAA positive	Sensitivity	Specificity
*LRRK2* p-syn-positive	1	1/1	100%	
*LRRK2* p-syn-negative	2	0/2		100%
*GBA* p-syn-positive	1	1/1	100%	
*GBA* p-syn-negative	3	0/3		100%
iPD p-syn-positive	10	10/10	100%	
iPD p-syn-negative	2	0/2		100%

## Discussion

Peripheral α-syn is considered a promising biomarker for PD diagnosis (9, 13, 69, 70), impacting peripheral tissues such as the skin, salivary glands, and gastrointestinal (GI) tract (71–74). Recent research, however, has detected α-syn and p-α-syn immunoreactivity in the GI tract of both PD patients and healthy controls, indicating potential concerns related to the specificity of α-syn deposition (75). Various issues, such as low biopsy yield, invasive procedures (76), and low sensitivity and specificity, may also limit the practical feasibility and clinical applicability of such tissues (74). In contrast, skin biopsies offer a viable diagnostic alternative due to their simplicity, cost-effectiveness, minimally invasive nature, and high specificity and sensitivity (9, 20). Liu et al. (77) demonstrated a specificity of 100% and sensitivity of 83.3% for the diagnosis of PD based on immunofluorescence analysis of 50-μm skin punch biopsies. Similarly, our research achieved a specificity of 100% and sensitivity of 72%, in alignment with the above studies. The morphological characteristics of p-α-syn deposits in the skin are similar to those of Lewy neurites found in the brain (69), underscoring the specificity and potential of skin biopsies in the identification of PD.

Similar cutaneous synucleinopathies have recently been detected in several PD patients with common genetic risk factors, including *SNCA*, *DJ-1*, *GBA*, and *LRRK2* (8, 10–13). To date, however, no premortem neuropathological studies on *CHCHD2* and *RAB39B* mutations have been reported. In this study, we identified p-α-syn deposits in the skin nerve of a PD patient with the *CHCHD2* T61I mutation. Thus, we propose that cutaneous synucleinopathies are highly similar between PD patients with *CHCHD2* T61I mutations and those with iPD. As expected, p-α-syn was found predominantly in the cutaneous autonomic structures of the study patients. According to previous reports, increased α-syn deposition is associated with increased autonomic dysfunction, which may explain the symptoms of autonomic nerve dysfunction in patients carrying the *CHCHD2* T61I mutation (78). Recent brain autopsy of a PD patient with the *CHCHD2* T61I mutation revealed widespread Lewy pathology based on anti-p-α-syn immunostaining, including diffuse LBs and LNs (16). Thus, these results indicate that peripheral cutaneous synucleinopathy is consistent with brain autopsy pathology in PD patients harboring the *CHCHD2* T61I mutation.

At present, the role of *RAB39B* in PD remains poorly understood. In the current study, p-α-syn deposition was not detected in the skin nerve fibers of PD patients carrying the *RAB39B* E179fsX48 mutation. Nonetheless, neuropathological features of PD, including neuronal loss and Lewy pathology in the SN and cortex, have been reported in PD patients with the *RAB39B* T168K mutation (17, 18). A recent theoretical framework suggests two PD progression models: a “brain-first” scenario where initial pathological α-syn emergence occurs within the central nervous system, starting from the SNpc and spreading through interconnected structures to the autonomic nervous system; and a “body-first” scenario where pathological α-syn begins in the enteric nervous system, then advances caudo-rostrally to the autonomic and central nervous systems (79, 80). In this study, brain autopsy findings from PD patients with *RAB39B* mutations revealed α-syn deposition in the SNpc and cortex but not in skin biopsies. Thus, we propose that PD patients with *RAB39B* mutations may predominantly exhibit a “brain-first” progression pattern.

Skin biopsies can yield insights into peripheral pathologies; yet the degree to which these observations accurately reflect intracranial pathologies requires further research, with few relevant reports on this topic. In this study, we compared synucleinopathy characteristics across genetic PD patients identified via skin biopsies and brain autopsies, considering mode of inheritance, genotype, ethnicity, and α-syn deposition. Notably, α-syn deposition was present in all skin biopsies and brain autopsies of AD-PD cases (involving *CHCHD2*, *LRRK2*, *SNCA*, and *GBA* mutations), suggesting a concordance between cutaneous and brain synucleinopathies in AD-PD patients. For AR-PD patients, α-syn deposition was observed in both skin biopsies and brain autopsies in *DJ-1*-mutant PD patients, whereas synucleinopathy was observed only in brain autopsies in *PRKN*-mutant PD patients. This discrepancy in the incidence of AR-PD may be linked to *PRKN* mutations leading to nigral degeneration without Lewy pathology (63, 68, 81), a pattern confirmed by our skin biopsy results from 16 *PRKN* mutation carriers. However, despite the absence of Lewy pathology, synucleinopathies have still been observed in brain autopsies of older PD patients with *PRKN* mutations (6, 7, 63–65). Several hypotheses have been posited to explain synucleinopathy in PRKN-associated PD, including: (i) a gradual accumulation of α-syn with aging in these patients; (ii) a diminished capacity in late-onset PD patients to clear accumulated proteins; and (iii) the possibility that mutations result in only a partial loss of function in the PRKN ubiquitin E3 ligase (63). Consequently, the presence of cutaneous synucleinopathy aligns closely with brainstem synucleinopathy in genetic PD, particularly among AD-PD cases.

Each gene encompasses multiple genotypes, with various mutation sites influencing functional outcomes and behavior of downstream proteins, leading to diverse phenotypes. In this study, identical mutation sites in *CHCHD2* (T61I) (16), *LRRK2* (G2019S, R1441G) (3, 30, 31), *SNCA* (E46K, A53T, duplication) (8, 40, 41), and *GBA* (N370S, E326K, L444P, N409S/D409H) (4, 5, 10, 52–56) were identified in both skin biopsies and brain autopsies, with synucleinopathy detected in both cutaneous and cerebral samples. These results indicate that the same genotypes contribute to similar synucleinopathic changes in the central nervous system and peripheral skin. Conversely, skin biopsies and brain autopsies did not share mutation sites in *DJ-1* (12, 62), *PRKN* (6, 7, 11, 30, 63–65, 67, 68), or *RAB39B* (17, 18). Notably, we found Lewy body pathology in the brain autopsies of patients with the *PRKN* R275W point mutation. The R275W mutation, located in RING finger 1 of the parkin protein (amino acids 238–293), alters protein distribution, leading to significant cytoplasmic and nuclear inclusions (82). Patients with the *PRKN* R275 W mutation tend to have an earlier AAO and greater severity of disease than patients with two truncating mutations, suggesting a dominant negative effect (82). Cookson et al. (82) demonstrated that the intracellular inclusion bodies are aggresomes and a cellular response to misfolded proteins in primary cultured neurons. However, no α-syn deposits have been previously detected in PD patients with compound heterozygous R275 W/E3-4 deletion or R275 W/Pro113fs in skin biopsies and brain autopsies (30, 66), hinting at the possibility that another mutation site may offset the negative effects of R275W—a hypothesis that merits further research.

To some extent, certain gene mutations display ethnic specificity. In this study, the *CHCHD2* T61I mutation was exclusively detected in the skin biopsies and autopsies of Asian individuals, suggesting a potential Asian-specific prevalence of the T61I genotype. Similarly, the *LRRK2* G2385R mutation, which is strongly associated with PD in Asian populations (83), was observed in both skin biopsies and brain autopsies of Asian individuals. Furthermore, skin biopsy synucleinopathy associated with the *RAB39B* E179fsX48 mutation was found solely in Asians, similar to that observed with *CHCHD2*, which needs further verification. Hence, our findings suggest that the *CHCHD2* T61I, *LRRK2* G2385R, and *RAB39B* E179fsX48 genotypes may predominantly occur in those with Asian ethnicity. These findings underscore the potential for the discovery of more ethnicity-specific genotypes related to cutaneous synucleinopathies, particularly within Asian cohorts, in future studies.

As a widely distributed neuronal protein, α-syn is highly enriched in presynaptic nerve terminals. The accumulation of misfolded oligomers and aggregates of α-syn, indicative of PD and other neurodegenerative synucleinopathies (84), was observed within the somatosensory and autonomic nerves in skin biopsies of PD patients with *CHCHD2*, *LRRK2*, and *GBA* mutations in our study. Comparative immunohistochemical analyses highlighted 5G4 as an effective marker, revealing more extensive and distinct α-syn pathology compared to other aggregates. α-Synuclein oligomers are known to disrupt intracellular trafficking, elevate intracellular calcium levels, and lead to synaptic dysfunction and loss (85). The detection of α-syn oligomers and aggregates in the peripheral nerves of skin biopsies aligns with the findings of our literature review of postmortem brain tissue in genetic PD patients.

In neurodegenerative diseases, especially in PD, intracellular α-syn and tau aggregates are commonly observed together, indicating co-neuropathology. The tau protein is subjected to extensive post-translational modifications, such as phosphorylation, deglycosylation, and truncation, resulting in insoluble, misfolded, and aggregated protein isoforms (86). Notably, tau inclusions have been identified within nigral neurons of partially purified Lewy bodies, with some studies reporting tau inclusions in 50% of PD brains (87). In this study, we detected tauopathies containing phosphorylated tau (AT8) and tau40 (HT7) deposited in skin nerves. Furthermore, tauopathies were observed in PD patients with *RAB39B* and *PRKN* mutations prior to the development of synucleinopathies, suggesting that tau accumulation may be upstream of α-syn aggregates. Thus, our findings on the peripheral cutaneous characteristics of genetic PD are consistent with previous reports that identified pathological tau as an early pre-synuclein process of nigrostriatal degeneration in premotor PD (86).

In this study, the analysis of α-syn using SAA in skin biopsies showed unparalleled sensitivity and specificity for detecting iPD and PD in individuals with mutations in *LRRK2* G2385R and *GBA*. Originally developed for prion disease detection, the application of SAA to skin tissue facilitates seeding activity assessment. Recent applications on postmortem brain and CSF have successfully identified positive α-syn in patients with PD associated with the *LRRK2* G2019S mutation and Lewy body pathology, but not in those with the *LRRK2* R1441G mutation (88). Therefore, prion-like seeding activity in peripheral skin may serve as an indicator of brain neuropathology in iPD and in certain cases of genetic PD. Consequently, α-syn SAA presents as a promising method for *in vivo* investigation of neuropathology in genetic PD.

Two research teams proposed biologically based staging systems for Parkinson’s disease (PD) around the same time. One system, named the neuronal α-syn disease integrated staging system (NSD-ISS) (89); The other approach, known as SynNeurGe, reflects the complexity and heterogeneity of PD using a three-component system (90). Both concepts are supported by advances in biomarkers, allowing for the accurate detection of pathological α-syn in tissue or CSF using SAA. Our discovery of peripheral cutaneous synucleinopathy in genetic PD cases provides further evidence to support these staging systems.

One of the primary limitations of this study is its small sample size. However, given the rarity of genetic PD and its significance in exploring pathogenesis of PD, our research still provides valuable insights into peripheral cutaneous synucleinopathy in individuals with iPD and genetic PD. Another limitation of this study is the absence of synucleinopathic data from both skin biopsies and autopsy samples for the same genetic PD patient, complicated by cultural considerations that impact the feasibility of performing autopsies in Asian populations. Future studies, incorporating long-term observations of genetic PD cohorts, are needed to obtain in-depth clinical data and speculate on the possible pathogenesis of this disease.

In conclusion, our study of peripheral cutaneous synucleinopathy characteristics in patients with genetic PD yielded several key findings: (i) P-α-syn was deposited in the peripheral cutaneous nerves of PD patients with *CHCHD2*, *LRRK2*, or *GBA* mutations but not in those with *RAB39B* or *PRKN* mutations. (ii) The distribution and frequency of α-syn-positive deposits did not significantly differ between genetic PD and iPD patients. (iii) Peripheral cutaneous synucleinopathy closely mirrored the intracranial synucleinopathy observed in genetic PD, especially in AD-PD. (iv) The detection of peripheral cutaneous synucleinopathy through RT-QuIC may enhance diagnostic precision for PD diagnosis in future applications. Thus, peripheral skin biopsies may serve as an effective approach for identifying biomarkers of iPD and genetic PD. Further studies on cutaneous synucleinopathy in genetic PD patients should help in elucidating the pathophysiology of genetic PD and developing precise therapeutic interventions.

## Data availability statement

The original contributions presented in the study are included in the article/Supplementary material, further inquiries can be directed to the corresponding authors.

## Ethics statement

The studies involving humans were approved by the Ethics Committee of the First Affiliated Hospital of Zhengzhou University. The studies were conducted in accordance with the local legislation and institutional requirements. The participants provided their written informed consent to participate in this study. Written informed consent was obtained from the individual(s) for the publication of any potentially identifiable images or data included in this article.

## Author contributions

YY: Supervision, Software, Methodology, Investigation, Funding acquisition, Formal analysis, Data curation, Conceptualization, Writing – review & editing, Writing – original draft. YW: Validation, Writing – original draft. ML: Writing – original draft, Methodology, Investigation. HL: Writing – original draft, Methodology, Data curation. XL: Writing – original draft, Methodology, Investigation. LL: Writing – original draft, Formal analysis, Data curation. CM: Writing – original draft, Investigation, Data curation. TY: Writing – original draft, Methodology. SL: Writing – original draft, Methodology. XZ: Writing – original draft, Funding acquisition. YG: Writing – original draft, Supervision. YX: Writing – review & editing, Visualization, Supervision, Software, Funding acquisition. JY: Writing – review & editing, Supervision, Software, Methodology, Funding acquisition.
