# Correction: Peripheral cutaneous synucleinopathy characteristics in genetic Parkinson's disease

**DOI:** 10.3389/fneur.2025.1677077

**Published:** 2025-08-21

**Authors:** Yanpeng Yuan, Yangyang Wang, Minglei Liu, Haiyang Luo, Xiaojing Liu, Lanjun Li, Chengyuan Mao, Ting Yang, Shuo Li, Xiaoyun Zhang, Yuan Gao, Yuming Xu, Jing Yang

**Affiliations:** ^1^Department of Neurology, The First Affiliated Hospital of Zhengzhou University, Zhengzhou, Henan, China; ^2^Henan Key Laboratory of Cerebrovascular Diseases, Zhengzhou University, Zhengzhou, Henan, China; ^3^Institute of Neuroscience, Zhengzhou University, Zhengzhou, Henan, China; ^4^NHC Key Laboratory of Prevention and Treatment of Cerebrovascular Disease, Zhengzhou, Henan, China

**Keywords:** skin biopsy, genetic Parkinson's disease, SAA, α-synuclein, *CHCHD2*, *RAB39B*

In the published article, there was an error in the Funding statement. A fund listed in the funding statement was corrected from “National Natural Science Foundation of China to JY (81600946)” to “National Natural Science Foundation of China to JY (82171434).”

The correct funding statement appears below.

