# Supplementary material for: Peripheral cutaneous synucleinopathy characteristics in genetic Parkinson’s disease
Source: Front Neurol. 2024 May 1;15:1404492. doi: 10.3389/fneur.2024.1404492 (PMC11094647; doi:10.3389/fneur.2024.1404492)
Supplement: Supplementary file 1 [file Table_1.DOCX]

Table S1 Immunofluorescence antibody list

| Antibody | Cat log | Dilution | Host | Monoclonal/ polyclonal | Vendor |
| --- | --- | --- | --- | --- | --- |
| PGP9.5 | MCA4750GA | 1:2000 | mouse | monoclonal | Bio-Rad |
| PGP9.5 | ab108986 | 1:2000 | rabbit | monoclonal | Abcam |
| p-syn | Ab51253 | 1:500 | rabbit | monoclonal | Abcam |
| α-synuclein | AB5038 | 1:500 | mouse | polyclonal | Millipore |
| 5G4 | MABN389 | 1:500 | mouse | monoclonal | Millipore |
| ASyO5 | AS13 2718 | 1:400 | mouse | monoclonal | Agrisera |
| TH | MAB318 | 1:100 | rabbit | monoclonal | Millipore |
| CGRP | ab81887 | 1:400 | rabbit | monoclonal | Abcam |
| VIP | sc-25347 | 1:100 | mouse | monoclonal | Santa Cruz |
| AT8 | MN1020 | 1:400 | mouse | monoclonal | Invitrogen |
| HT7 | MN1000 | 1:500 | mouse | monoclonal | Invitrogen |
| TDP43 | ab109535 | 1:400 | rabbit | monoclonal | Abcam |
| Ubiquitin | ab7780 | 1:500 | rabbit | polyclonal | Abcam |
| Aβ40 | NBP144047 | 1:400 | rabbit | polyclonal | Novus |
| Aβ42 | Ab10148 | 1:200 | rabbit | monoclonal | Abcam |
